# Ischemic postconditioning and pinacidil suppress calcium overload in anoxia-reoxygenation cardiomyocytes via down-regulation of the calcium-sensing receptor

**DOI:** 10.7717/peerj.2612

**Published:** 2016-11-01

**Authors:** Lin Zhang, Song Cao, Shengli Deng, Gang Yao, Tian Yu

**Affiliations:** 1 Department of Anesthesiology, Zunyi Medical College, Zunyi, Guizhou, China; 2 Guizhou Key Laboratory of Anesthesia and Organ Protection, Zunyi Medical College, Zunyi, Guizhou, China

**Keywords:** Ischemia-reperfusion (I/R) injury, Postconditioning, Calcium-sensing receptor (CaSR), ATP sensitive potassium channel (KATP)

## Abstract

Ischemic postconditioning (IPC) and ATP sensitive potassium channel (KATP) agonists (e.g. pinacidil and diazoxide) postconditioning are effective methods to defeat myocardial ischemia-reperfusion (I/R) injury, but their specific mechanisms of reducing I/R injury are not fully understood. We observed an intracellular free calcium ([Ca^2+^]_i_) overload in Anoxia/reoxygenation (A/R) cardiomyocytes, which can be reversed by KATP agonists diazoxide or pinacidil. The calcium-sensing receptor (CaSR) regulates intracellular calcium homeostasis. CaSR was reported to be involved in the I/R-induced apoptosis in rat cardiomyocytes. We therefore hypothesize that IPC and pinacidil postconditioning (PPC) reduce calcium overload in I/R cardiomyocytes by the down-regulation of CaSR. A/R model was established with adult rat caridomyocyte. mRNA and protein expression of CaSR were detected, IPC, PPC and KATP’s effects on [Ca^2+^]_i_ concentration was assayed too. IPC and PPC ameliorated A/R insult induced [Ca^2+^]_i_ overload in cardiomyocytes. In addition, they down-regulated the mRNA and protein level of CaSR as we expected. CaSR agonist spermine and KATP blocker glibenclamide offset IPC’s effects on CaSR expression and [Ca^2+^]_i_ modulation. Our data indicate that CaSR down-regulation contributes to the mitigation of calcium overload in A/R cardiomyocytes, which may partially represents IPC and KATP’s myocardial protective mechanism under I/R circumstances.

## Introduction

Strategies to limit myocardial ischemia-reperfusion (I/R) injury have not been well applied in clinical settings. Ischemic postconditioning (IPC) has been proved to be as effective as ischemic preconditioning in reducing infarct size, creatine kinase and preserving endothelial function in I/R hearts (Staat et al., 2005; Zhao et al., 2003).

ATP sensitive potassium channel (KATP) were first described by Noma in cardiac ventricular myocytes (Noma, 1983). Since then, pharmacological studies showed that KATP openers exerted profound cardioprotective effects in numerous mammalian species (Afzal et al., 2016; Flagg et al., 2010; Gao et al., 2016; Grover & Garlid, 2000; Yamada et al., 2006; Zingman et al., 2002). Following the finding of the mitochondrial KATP that locating at the inner membrane of mitochondria in 1991 (Inoue et al., 1991), Garlid et al. (1997) and Liu et al. (1998) demonstrated it as a trigger of ischemic preconditioning. Ischemic myocardium protection have been achieved by drugs such as pinacidil and diazoxide that open KATP (Garlid et al., 1997; Garlid et al., 1996). Instead, KATP blockers (5-hydroxydecanote or glibenclamide) cancelled the benefits of preconditioning and pharmacological treatments (Garlid et al., 1997; Gross, 1995; Liu et al., 1998). It’s also demonstrated that pharmacologically inhibition of KATP in early reperfusion abolished the infarct-limiting effects of IPC (Donato et al., 2007; Mykytenko et al., 2008; Yang et al., 2004). To date, the possible mechanisms of KATP in I/R hearts were various: swelling of mitochondria, increased fatty acid oxidation, ATP production and mitochondrial respiration in heart (Halestrap, 1989); inhibition of ATP hydrolysis during ischemia (Belisle & Kowaltowski, 2002; Dzeja et al., 2003); preservation of ATP and reduction of Ca^2+^ overload in caydiomyocytes (Cao et al., 2015).

Calcium-sensing receptor (CaSR) regulates systemic calcium homeostasis in several organs and tissues (Hu et al., 2014a; Lee et al., 2012). In 2003, Wang et al. (2003) first reported that CaSR existed in rat heart. As a G-protein coupled receptor in cardiomyocytes, CaSR is able to increase the concentration of IP3 by activating phospholipase C (Wang et al., 2006; Wang et al., 2003). CaSR also caused Ca^2+^ releasing from the sarcoplasmic reticulum (SR) into the mitochondria, which induced apoptosis of cardiomyocytes through the SR and mitochondrial related apoptotic pathway (Lu et al., 2013). CaSR activation aggravated the apoptosis of cardiomyocytes in diabetic rats by inducing calcium overload and activating mitochondrial pathway (Qi et al., 2013). It’s even reported that during cardiac I/R process, CaSR was over-expressed, which was involved in the calcium overload induced cardiomyocyte apoptosis (Zhang et al., 2006). Although CaSR activation during ischemic preconditioning may be myocardial protective in mice (Sun & Murphy, 2010), it has been well documented that IPC achieved myocardium protection partially by CaSR inhibition (Dong et al., 2010; Gan et al., 2012).

Our previous studies showed that artificially open KATP, either the mitochondrial KATP (Cao et al., 2015; Cao et al., 2016) or both of the sarcolemmal and mitochondrial KATPs (Yang & Yu, 2010; Yang et al., 2016), effectively reduced intracellular free calcium ([Ca^2+^]_i_) overload and cardiac I/R injury. Pinacidil is a nonselective KATP opener, which provided obvious myocardial protective effects when it was added in the preservation solution of rat heart (Yang & Yu, 2010). In addition, pinacidil postconditioning (PPC) has recently been proven to be protective in I/R hearts, and PPC’s effects was comparative to that of IPC (Yang et al., 2016). Nevertheless, our understanding of its specific mechanism, and the correlations among KATP, IPC and [Ca^2+^]_i_ overload in I/R heart remained quite preliminary.

In this study, we characterized an anoxia/reoxygenation (A/R) model using acutely isolated adult rat cardiomyocytes. KATP status was interfered with its specific opener pinacidil or blocker glibenclamide. To test the hypothesis that IPC and PPC reduce calcium overload in A/R heart by down-regulating CaSR, mRNA and protein levels of CaSR will be detected in rat cardiomyocytes, and the relationship between KATP and CaSR will be examined too.

## Material and Methods

### Animals

Male Sprague-Dawley rats (250–300 g, 16–20 weeks) were provided by the Third Military Medical University (Chongqing, China) and maintained in specific pathogen free (SPF) animal facility in Zunyi Medical College under standardized conditions with 12 h light/dark cycles (8:00 am–8:00 pm with light on) and free access to rat chow and water. All experimental procedures were performed in accordance with the Guide for the care and use of laboratory animals in China. Experiment procedures were also approved by the Experimental Animal Care and Use Committee of Zunyi Medical College (approval No. 2008115).

### Isolation of adult rat cardiomyocytes

When deeply anesthetized with sodium pentobarbital (60 mg/kg, combined with 250 U/kg heparin; intraperitoneal injection), the rat hearts were excised rapidly. Ventricular cardiomyocytes were obtained with enzymatic digestion method as previously described (Son et al., 2011). Briefly, rat hearts were retrogradely perfused with 0.1% type II collagenase at constant pressure (9 mL/min/g) on the Langendorff apparatus. Then the ventricle tissue was collected and digested by type II collagenase solution. The modified M199 medium (with 2 mM carnitine, 5 mM taurine, 2 mM glutamine, 0.8 mM EGTA, 5 mM creatine) was used for culture of cells. Three hours later, the culture medium was replaced to eliminate non-cardiomyocytes. Cell viability was confirmed with trypan blue exclusion assay.

### IPC and PPC of cardiomyocytes

**Figure 1:**
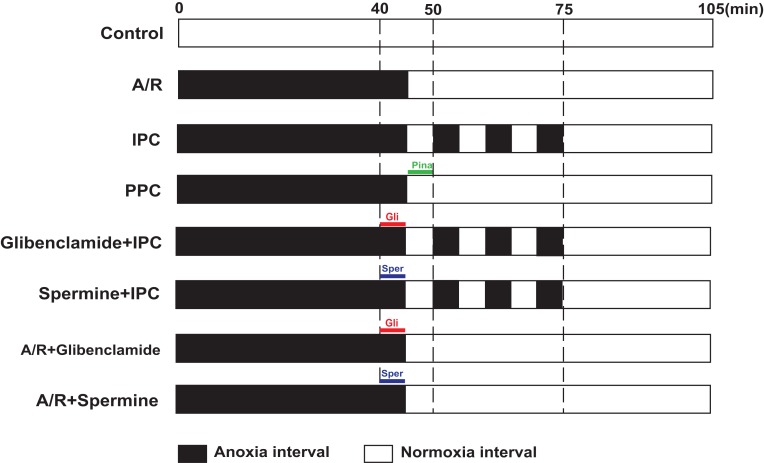
Illustration of A/R protocols. After 20 h culture in normoxic incubator, cardiomyocytes were randomly distributed to different groups. Cardiomyocytes of Control were continuously cultured in a normoxic incubator for 105 min. Medium of other groups was replaced with N_2_ bubbled (95% N_2_, 5% CO_2_) M199 for the first 45 min, then replaced with O_2_ bubbled M199. IPC group underwent three cycles of reoxygenation/anoxia (5 min: 5 min) before 30 min normal culture. Different concentration of pinacidil was added into the M199 and incubated with cardiomyocytes for 5 min at the beginning of reoxygenation in PPC groups. Glibenclamide + IPC group and spermine + IPC group were treated with 5 min glibenclamide (Gli) or spermine (Sper), respectively before IPC treatments (3 reoxygenation/anoxia (5 min: 5 min)). A/R + glibenclamide and A/R + spermine group were treated with 5 min glibenclamide or spermine, respectively at the end of anoxia periods before reoxygenation. Each protocol took 105 min in total.

The I/R model in adult cardiomyocytes was established as we previously reported (Cao et al., 2015). Cells were incubated in normoxic incubator for 20 h before randomly distributed to 10 groups: Control, A/R, IPC, PPC 10, PPC 30, PPC 100 μM, glibenclamide + IPC, spermine + IPC, A/R + glibenclamide and A/R + spermine group. Cardiomyocytes of Control were continuously cultured in a normoxic incubator for 105 min. Normoxic medium of other groups was replaced with N_2_ bubbled (95% N_2_, 5% CO_2_) modified M199 and incubated in an O_2_/CO_2_ incubator containing a humidified atmosphere of less than 1% O_2_, 5% CO_2_ and 94% N_2_ at 37 °C for the first 45 min, then replaced with O_2_ bubbled M199. IPC group underwent three cycles of reoxygenation/anoxia (5 min: 5 min) before 30 min normal culture. Different concentration of pinacidil was added into the M199 and incubated with cardiomyocytes for 5 min at the beginning of reoxygenation in PPC groups. Glibenclamide + IPC group and spermine + IPC group were treated with 5 min glibenclamide or spermine, respectively before IPC treatments (3 reoxygenation/anoxia (5 min: 5 min)). A/R + glibenclamide and A/R + spermine group were treated with 5 min glibenclamide or spermine, respectively at the end of anoxia periods before reoxygenation. Each protocol took 105 min in total (Fig. 1). Oxygen deprivation and reoxygenation were managed by series of changes of normoxic or anoxic medium (bubbled with O_2_ or N_2_) and incubators. Normal culture was conducted in a normoxic incubator (O_2_/CO_2_ incubator with 5% CO_2_ and 95% air at 37 °C).

### Intracellular calcium concentration ([Ca^2+^]_i_) detection

At the end of reoxygenation, [Ca^2+^]_i_ in cardiomyocytes was detected as previously reported (Zhang et al., 2006). Briefly, M199 was removed, cells of eight groups were washed twice with PBS before incubation with Fluo-3 AM (Biotium, Fremont, CA, USA) at a final concentration of 10 μM for 30 min at 37 °C. After incubation, cells were washed twice with PBS. The fluorescence intensity of Fluo-3 AM, which represented [Ca^2+^]_i_ concentration was detected using a TCS SP2 AOBS confocal microscope (Leica, Germany). The excitation and emission wavelength of Fluo-3 were set at 488 nm and 525 nm, respectively. More than 50 cells of each group were randomly selected for data analysis, their outlines were circled out manually and the fluorescence density of Fluo-3 AM was calculated with Leica Confocal software (Leica, Wetzlar, Germany).

### Cell viability detection

The viability of adult cardiomyocytes was detected with Cell Counting Kit-8 (CCK-8; Beyotime, Jiangsu, China) as we previous reported (Cao et al., 2015). Same amount of cells were seeded into 24-well plates. At the end point of reoxygenation, 30 μL WST-8 solution was added into M199 to form a 3% WST-8 final concentration. Cells were incubated for one hour before the mixture’s OD value was detected at 450 nm wavelength.

### RT-PCR

Real-time quantitative PCR (RT-PCR) was employed to detect mRNA expression change of CaSR and β-actin were selected as the reference gene. Total RNA was collected with TRIzol protocol using the TaKaRa RNAiso Kit (TaKaRa, Japan). RNA concentration and purity were checked using a Varioskan Flash spectrophotometer (Thermo Fisher, Waltham, MA, USA). 500 ng RNA was reversely transcribed to cDNA according to the manufacturer’s protocol using a cDNA synthesis kit (TaKaRa, Shiga, Japan) in a final volume of 10 μL. RT-PCR was performed with a CFX Connect Real-Time system (Bio-Rad, USA) using a SYBR green PrimScript RT kit (TaKaRa, Shiga, Japan). The PCR conditions included pre-denaturing at 95 °C for 2 min followed by 40 cycles of denaturation at 95 °C for 10 s and combined annealing/extension at 61 °C for 15 s. The expression levels were calculated based on the comparative quantification method (2^−ΔCT^). The CaSR (NM016996) primer sequences were: forward: 5′-TCGGCATCAGCTTTGTGCTC-3′, reverse: 5′-AAGCTGGTGGGTATCTTGGCTTC-3′; and the β-actin (NM031144) primer sequences were: forward: 5′-GGAGATTACTGCCCTGGCTCCTA-3′, reverse: 5′-GACTCATCGTACTCCTGCTTGCTG-3′. All of the primers were products of TaKaRa (Dalian, China).

### Western blot

Twenty microgram of protein from the adult cardiomyocytes was separated by 6% (CaSR) or 10% (β-actin) SDS-PAGE. The target proteins were transferred to polyvinylidene fluoride (PVDF) membranes, which were blocked overnight in TBST (20 mM Tris and 150 mM NaCl, pH 8.0) containing nonfat milk powder. Then membranes were probed with 1 mg/mL monoclonal primary antibodies (1:500 dilution) of CaSR (ab19347; Abcam,Cambridge, UK) or β-actin for 1 h. PVDF membranes were incubated with horseradish peroxidase-conjugated secondary antibody (1:500) for 1 h and then enhanced chemiluminescence (Amersham Biosciences, Piscataway, NJ, USA). Immunoreactivity was visualized by a ChemiDoc MP system (Bio-Rad, Hercules, CA, USA). Protein levels were normalized to β-actin. Optical density of the protein bands were measured after subtracting the film background.

### Statistical analysis

Data were expressed as mean ± SD. For comparisons among groups, one-way analysis of variance (ANOVA) was firstly performed; then a post hoc LSD or Dunnett’s T3 method was used. A P value of less than 0.05 was set as the statistically significant threshold. All analyses were carried out using SPSS (v.17, IBM, USA).

## Results

### Isolated adult rat cardiomyocytes

**Figure 2:**
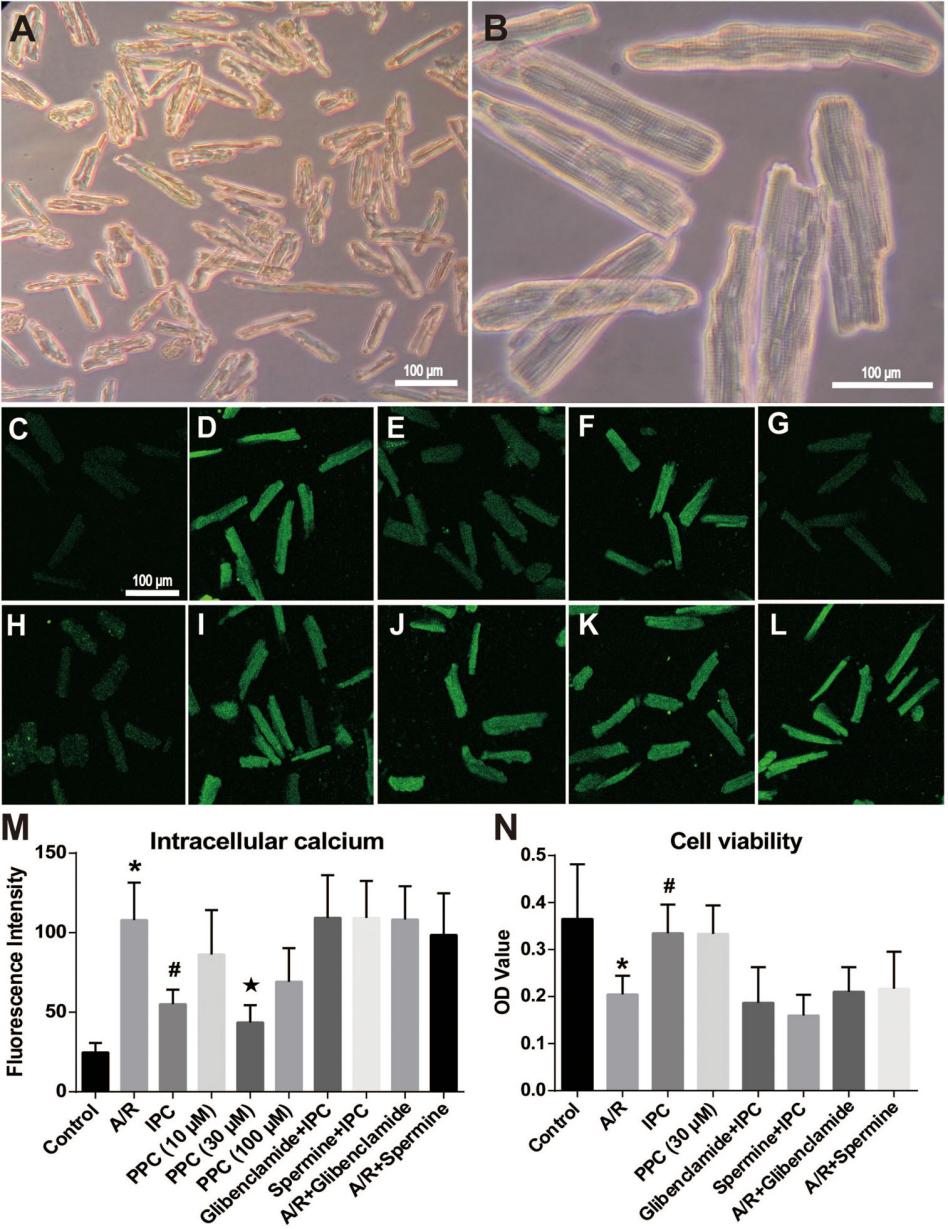
[Ca^2+^]_i_ and cell viability detection in acutely isolated rat cardiomyocytes after A/R, IPC, PPC, KATP blocker or CaSR agonist treatment. (A–B) The morphology of acutely isolated adult rat cardiomyocytes. The ventricular myocytes were rod-shaped, with clear cross striations. (C–L) The effect of different treatment on the [Ca^2+^]_i_ level in adult rat cardiomyocytes. At the end point of reoxygenation, cells of Control (C), A/R (D), IPC (E), PPC (10 μM) (F), PPC (30 μM) (G), PPC (100 μM) (H), glibenclamide + IPC (I), spermine + IPC (J), A/R + glibenclamide (K) and A/R + spermine (L) group were incubated in 10 μM Fluo-3-AM for 60 min at 37 °C and detected with a confocal microscope. (M) The [Ca^2+^]_i_ fluorescence intensity comparison. [Ca^2+^]_i_ in A/R group increased dramatically compared with the Control group (P < 0.01). IPC and 30 or 100 μM pinacidil reduced the [Ca^2+^]_i_ intensity. After glibenclamide administration, fluorescence intensity increased to the A/R group level. CaSR agonist spermine offset IPC’s effect on [Ca^2+^]_i_ level too. *, P < 0.01 compared with Control, IPC and PPC group. #, P < 0.01 compared with glibenclamide + IPC, spermine + IPC, A/R + glibenclamide or A/R + spermine group. ★, P < 0.01 compared with PPC (10 μM) or PPC (100 μM) group. Data are mean ± SD, n = 25 cells for each group. (N) CCK-8 assay showed that A/R insult significantly decreased the cell viability while IPC and 30 μM PPC reversed the decrease. Glibenclamide or spermine use can offset IPC’s effect on cell viability. *, P < 0.01 compared with Control, IPC and PPC group. #, P < 0.01 compared with glibenclamide + IPC, spermine + IPC, A/R + glibenclamide or A/R + spermine group. Data are mean ± SD, n = 6.

Rod shape adult cardiomyocytes with clear striations and sharp outlines were harvested (Figs. 2A and 2B). Trypan blue exclusion assay showed that 60–80% of them were of good viability.

### [Ca^2+^]_i_ detection

Fluo-3 AM was used as the intracellular free calcium probe to examine Ca^2+^ concentration in cardiomyocytes (Figs. 2C–2M). In Control group, the level of [Ca^2+^]_i_ was the lowest. Compared with Control, [Ca^2+^]_i_ increased significantly after A/R treatment (P < 0.05). After the applying of 30 or 100 μM, but not 10 μM pinacidil, [Ca^2+^]_i_ decreased significantly compared with A/R group. Thirty μM pinacidil is the most effective one to decrease [Ca^2+^]_i_ (Figs. 2G and 2M). There were apparent increases (P < 0.05) in glibenclamide + IPC and spermine + IPC groups compared with A/R group. It indicated that pinacidil (30 or 100 μM) strongly inhibited [Ca^2+^]_i_, while the CaSR agonist spermine remarkably increased the [Ca^2+^]_i_ levels in adult rat cardiomyocytes after I/R injury.

### Cell viability

For 30 μM pinacidil is most effective in inhibiting [Ca^2+^]_i_ increase in A/R cardiomyocytes, we chose this concentration in the subsequent experiments. CCK-8 assay showed that A/R insult significantly decreased the cell viability of cardiomyocytes (P < 0.01), while IPC and 30 μM PPC reversed the decrease (P < 0.01). Glibenclamide or spermine use can offset IPC’s effect on cell viability (all P < 0.01, Fig. 2N).

### RT-PCR

**Figure 3:**
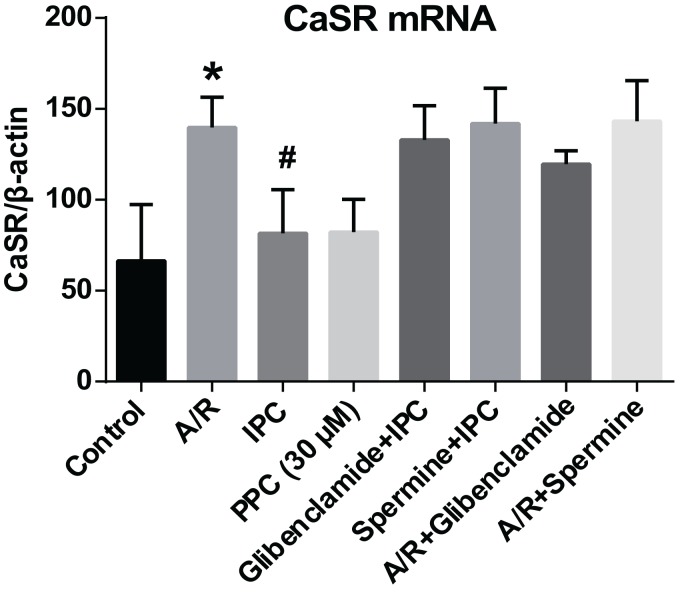
CaSR mRNA expression. At the end point of reoxygenation, cells of eight groups were subjected to RT-PCR to detect CaSR expression at the mRNA level. CaSR mRNA showed apparently increase in A/R group compared with the Control group. IPC and 30 μM pinacidil reduced this trend profoundly. After glibenclamide administration, CaSR expression increased nearly to the A/R group level. CaSR agonist spermine offset IPC’s effect on CaSR mRNA level too. Data are expressed as mean ± SD. n = 6 for each group. *, P < 0.01 compared with Control, IPC and PPC group. #, P < 0.01 compared with glibenclamide + IPC, spermine + IPC, A/R + glibenclamide or A/R + spermine group.

To evaluate the mRNA expression of CaSR, SYBR green based quantitative RT-PCR were carried out (Fig. 3). Control group showed the lowest CaSR expression level. The expression of CaSR increased significantly after A/R treatment (P < 0.05). After the applying of 30 μM pinacidil, CaSR mRNA amount decreased dramatically compared with A/R. There were apparent increases (P < 0.05) in glibenclamide + IPC and spermine + IPC groups compared with A/R group (Fig. 3). It indicated that IPC and PPC (30 μM) both strongly inhibited the CaSR expression at the mRNA level in adult rat cardiomyocytes, while the CaSR agonist spermine and KATP blocker glibenclamide offset IPC’s effects.

### Western blot

**Figure 4:**
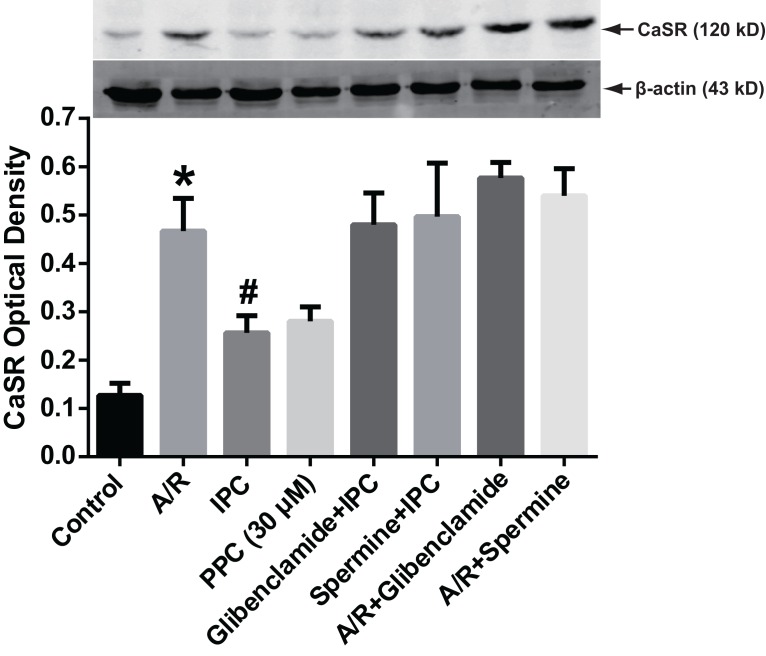
CaSR protein expression. At the end point of reoxygenation, cells were subjected to Western blot to detect CaSR ptotein expression change after different treatments. CaSR protein level increased dramatically after A/R insult compared with the Control group (P < 0.01). IPC and 30 μM pinacidil supressed CaSR over-expression after I/R injury. After the administration of glibenclamide or spermine, much of IPC’s suppression effects on CaSR expression were diminished. Data are mean ± SD. Three replicates for each group. *, P < 0.01 compared with Control, IPC and PPC group. #, P < 0.01 compared with glibenclamide + IPC, spermine + IPC, A/R + glibenclamide or A/R + spermine group.

To evaluate the protein expression of CaSR, we employed Western blot to detect the expression change at the protein level (Fig. 4). The Western blot data (normalized to β-actin) showed that compared with the Control group, the CaSR protein increased remarkably in A/R group. Significant difference existed between A/R and PPC (30 μM) group, after the postconditioning with 30 μM pinacidil, CaSR decreased dramatically compared with A/R group. Apparent increase of CaSR protein (P < 0.05) in glibenclamide + IPC and spermine + IPC group were also detected compared with A/R group.

## Discussion

Myocardial I/R injury is one of the leading causes of morbidity. [Ca^2+^]_i_ overload during I/R injury is the trigger of cell damage. In the present study, we found A/R dramatically increased [Ca^2+^]_i_ overload in isolated adult rat ventricular cells as we previously found (Cao et al., 2015). The [Ca^2+^]_i_ overload after reoxygenation can be alleviated by two kinds of postconditionings applied on cardiomyocytes, the IPC and PPC.

To test our hypothesis that [Ca^2+^]_i_ overload in I/R cardiomyocytes is (partially) resulted from CaSR over-expression, and the myocardial protective effects of IPC and PPC are (partially) contributed by down-regulation of CaSR. We detected the expression of CaSR in vitro at the end of reoxygenation. As we expected, CaSR mRNA and protein expression levels significantly increased when the adult rat cardiac cells receive A/R insult, which have been reported by Zhang et al. (2006).

Many studies indicated that KATP was the end effector of many cardiac protective strategies, such as the ischemic preconditioning (Brennan et al., 2015), remote preconditioning (Hu et al., 2014b) and exercise (Kraljevic et al., 2015). KATP also contributed to ischemic myocardium protection effects of IPC. Pharmacological inhibition of the KATP at the beginning of reperfusion abolished the infarct-limiting effects of IPC (Donato et al., 2007; Mykytenko et al., 2008; Yang et al., 2004). Therefore, we tested the involvement of KATP in the IPC settings. In addition, we directly interfered with KATP with its agonist pinacidil and inhibitor glibenclamide. Both the IPC and PPC suppressed [Ca^2+^]_i_ overload after A/R treatment. In addition, at the end of reoxygenation, both of the postconditoning methods leaded to the down-regulation of CaSR, characterized as decreased mRNA and protein level. IPC and PPC’s effects on [Ca^2+^]_i_ overload and CaSR expression were disappeared when cells received glibenclamide (KATP blocker) or spermine (CaSR agonist) treatments before the postconditioning.

Schreckenberg et al. (2015) found down-regulation of the CaSR by siRNA apparently affect electrical stimuli induced adult cardiomyocyte shortening in rat. siRNA mediated silencing of CaSR also alleviated high glucose induced rat cardiomyocyte injury, evidenced by increased [Ca^2+^]_i_, increased cardiomyocyte apoptosis, up-regulation of Bax, p-ERK, p-JNK and suppressed Bcl-2 expression in vitro (Qi et al., 2013).

Given that IPC or 30 μM PPC treatment strongly inhibited the CaSR expression at the mRNA and protein level in adult rat cardiomyocytes, while the CaSR agonist spermine and KATP blocker glibenclamide offset IPC’s effect. We therefore conclude that [Ca^2+^]_i_ overload in A/R cardiomyocytes is partially contributed by the up-regulation of CaSR in the anoxia period, which leads to the amplification of calcium induced [Ca^2+^]_i_ release in the reoxygenation interval. This trend can be partially suppressed by KATP, which also takes part in IPC and PPC’s myocardial protective mechanism. These results indicate that the opening of KATP is one of the downstream effects of CaSR down-regulation. We previously found that KATP opening kept the ATP homeostasis in adult cardiomyocytes (Cao et al., 2015), which could be the indirect protective mechanism of CaSR down-regulation. KATP also reported to be linked to the regulation of mitochondrial permeability transition pore (MPTP). It was proposed that the regulation of MPTP is involved in the cardioprotection by IPC (Mykytenko et al., 2008). These findings suggest that CaSR is a trigger of the increase of [Ca^2+^]_i_ in I/R settings. CaSR manipulation could be a potential strategy in defeating cardiac I/R injury.

We must confess that our study has some shortcomings. In vivo studies are warranted to further confirm CaSR’s effects on cardiac I/R injury (e.g. the [Ca^2+^]_i_ overload in cardiomyocytes) and its interaction with KATP. Besides CaSR agonist, direct genetic or post-translational manipulation of CaSR could be useful to make sure that IPC and KATP’s myocardial protective effect resulted from CaSR down-regulation.

## Supplemental Information

10.7717/peerj.2612/supp-1 Supplemental Information 1 Raw data: numerical values of bar charts in Figs. 2–4.

## Additional Information and Declarations

### Data Deposition

The following information was supplied regarding data availability: The raw data has been supplied as Supplemental Dataset Files.
